# Plantar-pressure-guided support-plate position control for a walker-assisted lower-limb rehabilitation exoskeleton

**DOI:** 10.3389/fbioe.2026.1911004

**Published:** 2026-09-03

**Authors:** Yuhui Yang, Wenyu Zhao, Yuanxiang Guo, Zhijue Huang, Xueshan Gao, Xin Han, Junlin Deng

**Affiliations:** 1 College of Mechanical and Marine Engineering, Beibu Gulf University, Qinzhou, China; 2 School of Automation, Guangxi University of Science and Technology, Liuzhou, China; 3 School of Mechatronics Engineering, Beijing Institute of Technology, Beijing, China

**Keywords:** compliant support, fixed-stiffness spring, initial-contact impact, lower-limb rehabilitation exoskeleton, measured actuator electrical energy, plantar-pressure zone fusion, support-force adjustment-plate position control

## Abstract

**Introduction:**

Maintaining appropriate foot-ground contact is important for walker-assisted lower-limb exoskeleton training. Excessive plantar loading may increase initial-contact impact and actuator load, whereas excessive unloading may reduce frictional contact and contact stability. Passive spring support can provide impact buffering, but it cannot adjust the support working point according to gait phase or plantar-pressure feedback.

**Methods:**

This study proposes a multi-zone plantar-pressure-guided position control method for the support-force adjustment plate of a walker-assisted lower-limb exoskeleton. The system combines a fixed-stiffness spring with a vertical screw-driven adjustment plate. Total plantar pressure was obtained by fusing signals from 18 pressure cells on each instrumented foot and served as the main feedback variable. Regional plantar pressures and the center of pressure supported gait-phase recognition and contact-state estimation. A phase-related plantar-pressure target band was used as an engineering constraint to maintain stable foot-ground contact. Experiments involved three healthy male participants under no-spring, passive-spring, and proposed-control conditions during low-speed frame-assisted walking.

**Results and discussion:**

In the proposed-control condition, peak total plantar pressure was 0.687±0.015 BW, 21.2% lower than in the no-spring condition and 6.7% lower than in the passive-spring condition. Relative to the no-spring condition, the loading rate decreased from 9.02±0.53 to 2.56±0.07 BW/s, and the initial-contact impact impulse decreased from 0.143±0.009 to 0.064±0.002 BW ⋅ s. Hip-knee actuator electrical energy decreased from 38.95±2.20 to 27.24±1.54 J/gait cycle. After vertical-axis actuator energy was included, measured total actuator electrical energy was 30.60±1.68 J/gait cycle and remained lower than in both comparison conditions. These preliminary results suggest that plantar-pressure feedback can regulate support-plate position, attenuate contact impact, and reduce measured robotic actuator effort during low-speed walker-assisted walking.

## 1 Introduction

Lower-limb rehabilitation exoskeletons are designed to provide repetitive, controllable, and quantifiable gait assistance for users with impaired walking function. Recent studies have advanced wearable and mobile lower-limb rehabilitation robots in mechanical design, trajectory tracking, interaction control, and assisted locomotion (Qiu et al., 2023; Jin and Guo, 2023; Yu et al., 2024). For users with weak trunk control or limited independent balance, a walker-assisted architecture provides an additional physical support boundary. This architecture improves operational safety, but it also changes how the human body, the exoskeleton, and the ground exchange load during stance. Therefore, a walker-assisted exoskeleton should be evaluated not only by joint motion tracking, but also by plantar-contact state, vertical support behavior, and actuator effort.

Body-weight support has been widely investigated as a way to reduce lower-limb loading during gait training. Cable-driven, mobile, and actively adjusted body-weight support systems can provide unloading and protection during overground or treadmill-based training (Li et al., 2023; Dong et al., 2021; Rodrigues and Goncalves, 2023; Qin et al., 2023; Choi, 2022). These systems show that vertical support can improve the feasibility of gait practice, but they also reveal a control trade-off. If support is purely passive, the spring or suspension can absorb part of the impact but cannot adapt its working point to gait phase or individual differences. If support is actively adjusted without contact-state feedback, the user may experience either insufficient unloading during stance or excessive unloading that weakens foot-ground contact (Park et al., 2022). This motivates a support mechanism that combines fast passive buffering with low-frequency position adjustment driven by plantar-pressure feedback.

Plantar pressure provides a direct measurement of the foot-ground contact outcome. The stance-phase plantar-pressure curve has a time-varying trajectory related to gait phase, rather than a single constant value (Wolff et al., 2023; Wolff et al., 2024). Modern pressure insoles commonly use multiple sensing cells, which makes it possible to calculate total plantar pressure, regional pressures, and the center of pressure (COP) after calibration (Ren and Liu, 2021; Conner et al., 2022). These signals have been used for gait-phase recognition, walking-speed estimation, abnormal-gait classification, and multimodal lower-limb state estimation (Liu et al., 2024; Cho, 2023; Jun et al., 2021; Ren et al., 2023). For control purposes, however, a single pressure cell is vulnerable to local contact changes and insole placement. A more robust strategy is to fuse all pressure cells into total plantar pressure while retaining heel, midfoot, forefoot, and COP information to describe load transfer.

Multi-sensor fusion and learning-based state estimation have also been introduced into lower-limb exoskeleton control. Integrated network models, compound networks, flexible pressure-sensor arrays, and limb-angle prediction methods have improved gait-phase recognition and lower-limb state estimation (Zhang et al., 2022; Xia et al., 2023; Zhou et al., 2025; Wang et al., 2025). Most of these studies use plantar pressure or inertial signals mainly as recognition inputs, while the outer-loop control objective remains focused on joint motion or joint assistance. In walker-assisted walking, plantar pressure should also be treated as a contact constraint. Excessively low plantar pressure can reduce the observable foot-ground contact state and weaken the pressure-based load-transfer information used for gait interaction (Wolff et al., 2023; Conner et al., 2022; Ren et al., 2023). Therefore, the controller in this study uses a phase-related pressure band, not a maximally unloaded or fixed pressure target.

Energy evaluation is another important issue in support-assisted exoskeleton control. Reducing hip and knee actuator load is beneficial only if the additional support actuator does not introduce a larger energy cost. Studies on assist-as-needed control, exoskeleton energy optimization, energy recycling, and measured actuator electrical energy show that actuator-level energy should be reported with a clear definition of voltage/current measurement and integration method (Wang et al., 2022a; Woo et al., 2021; Lee and Rosen, 2023; Andersson and Bjorsell, 2022; Tang et al., 2022; Chen et al., 2025). For the present system, the measured energy metric is limited to the hip, knee, and vertical-axis drive channels and is reported as measured actuator electrical energy.

This study therefore treats plantar pressure as a measurable contact constraint for support-plate position control in a walker-assisted exoskeleton. Beyond passive spring buffering, the proposed method adjusts the support working point using phase-related plantar-pressure feedback. The analysis accounts for vertical-axis actuator energy together with hip-knee actuator effort. Materials and Methods describes the robot, sensors, data processing, controller, and experimental protocol. The subsequent sections report plantar-pressure, joint-torque, and actuator-energy results and discuss their engineering implications for low-speed frame-assisted walking.

## 2 Materials and methods

### 2.1 Mechanical structure

**FIGURE 1:**
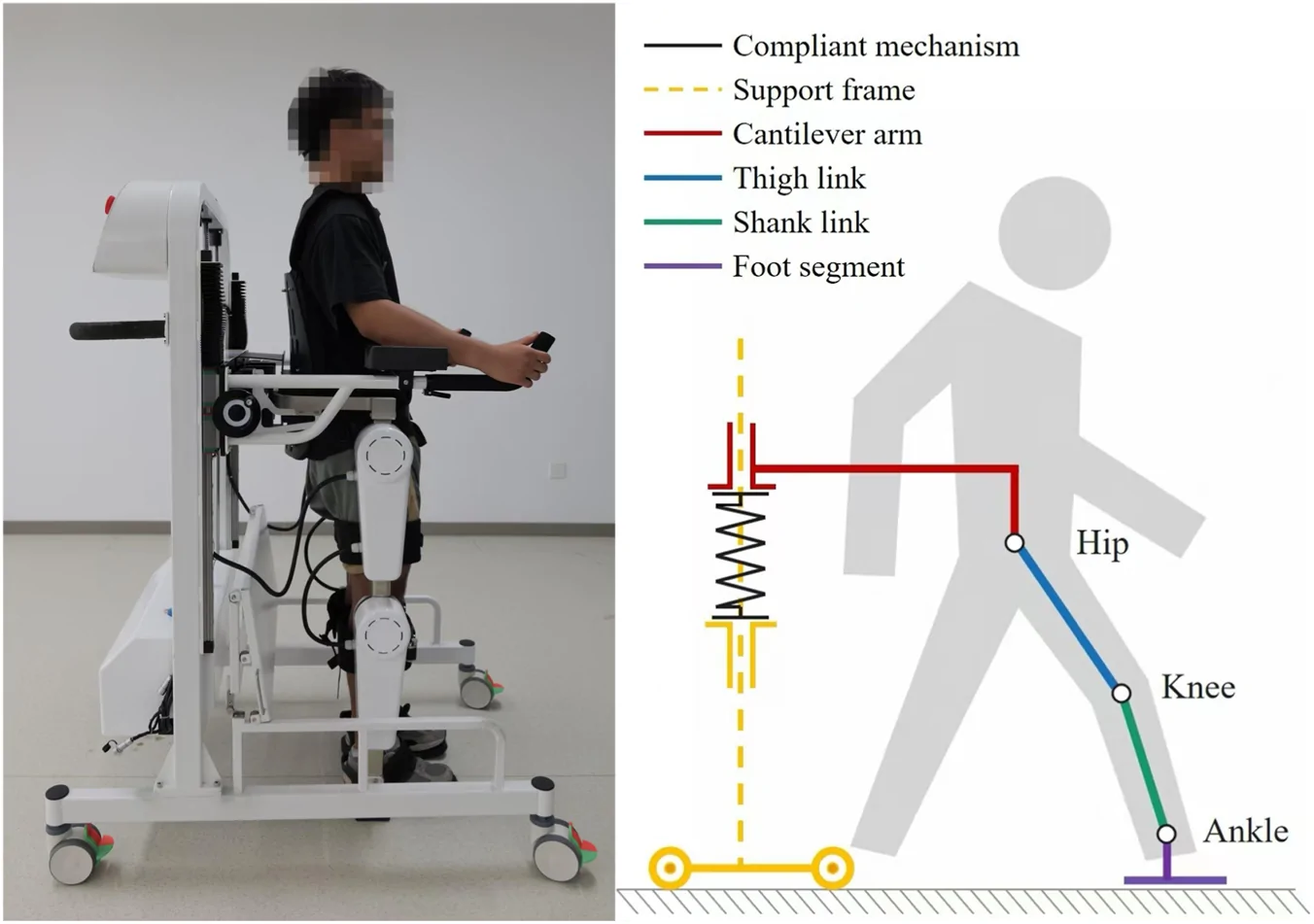
Overall mechanical structure of the walker-assisted lower-limb exoskeleton. The figure shows the support frame, cantilever arm, fixed-stiffness spring, thigh module, shank module, foot module, and experimental use scenario.

The robot consists of a walker frame, mobile wheels, a lower-limb exoskeleton body, hip and knee joint drive modules, a foot sensing module, a vertical screw motor, a support-force adjustment plate, and a fixed-stiffness spring (Figure 1). The vertical axis, denoted as the Z-axis in the hardware implementation, follows the direction of adjustment-plate travel. Upward plate motion increases the working compression of the fixed-stiffness spring, whereas downward motion decreases it. The joint modules actively assist hip and knee flexion/extension. The ankle is not actively actuated; the foot module provides mechanical connection and plantar-pressure sensing. One end of the spring connects to the support-force adjustment plate, and the other connects to the back plate or exoskeleton support structure. Spring stiffness remains fixed during operation, so the system is not a variable-stiffness mechanism.

This arrangement provides active-passive hybrid support. The fixed-stiffness spring supplies rapid passive buffering and absorbs part of the impact at foot contact. The vertical screw motor adjusts the support-force adjustment plate at low frequency to change the average unloading-related working point. Because the screw mechanism has limited adjustment speed, the vertical motor is not used to respond directly to high-frequency impact. Instead, plantar-pressure feedback adjusts the low-frequency support working point. This design follows the principle of combining rapid compliant buffering with low-frequency active adjustment in body-weight support and compliant exoskeleton control (Li et al., 2023; Dong et al., 2021; Rodrigues and Goncalves, 2023; Qin et al., 2023; Wang et al., 2022b; Hasan and Dhingra, 2022; Yu et al., 2023; Laubscher et al., 2023).

### 2.2 Sensor layout

**FIGURE 2:**
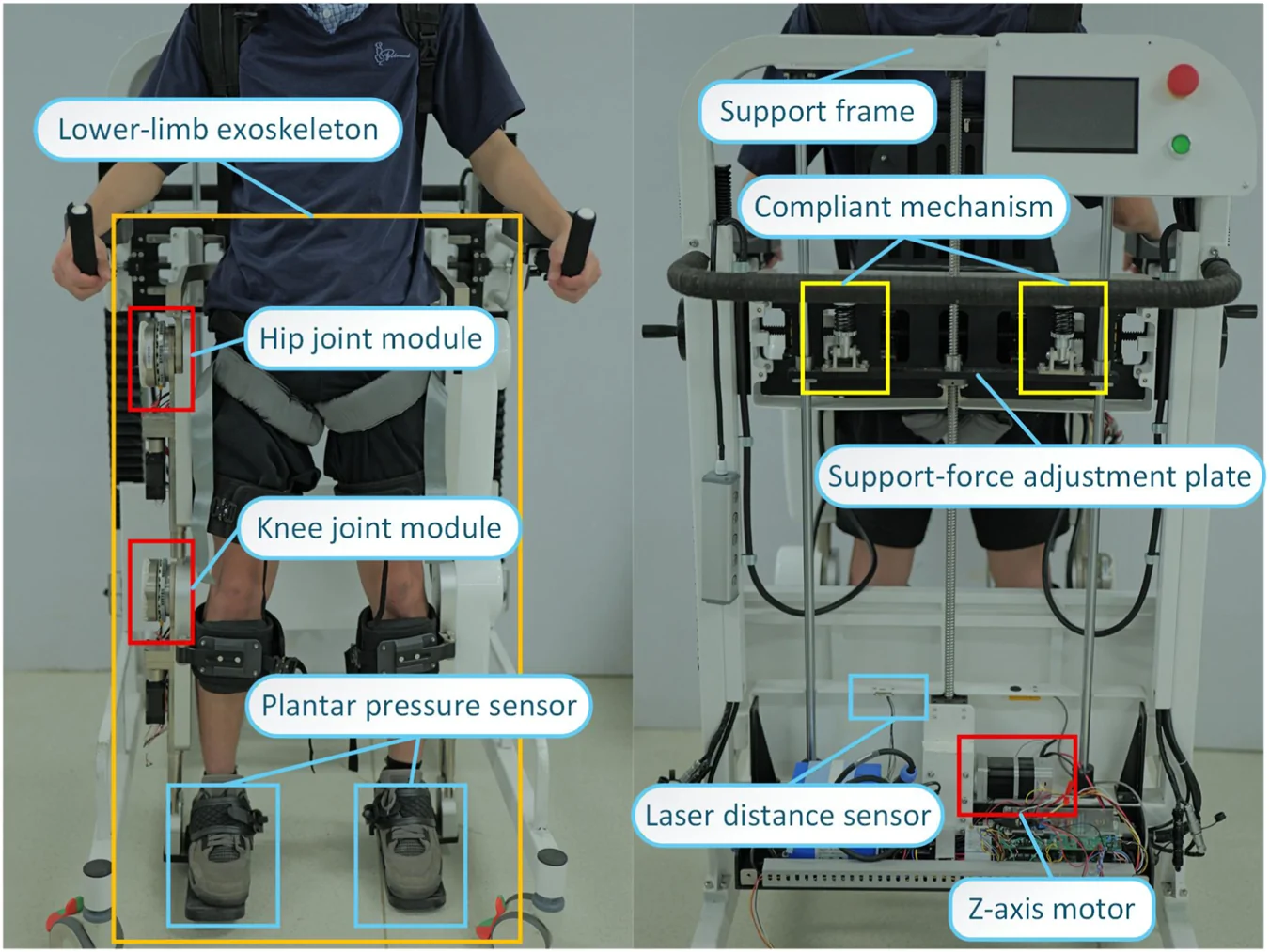
Prototype assembly and physical sensor layout. The figure marks the plantar-pressure sensor, hip/knee joint modules, laser distance sensor for support-force adjustment-plate position, vertical screw motor, and voltage/current acquisition channels.

The sensor system includes a multi-zone plantar-pressure sensor, dual encoders in the hip and knee joint modules, hip/knee motor voltage and current measurements, a laser distance sensor for adjustment-plate position, and vertical-axis motor voltage and current measurements. As shown in Figure 2, the laser sensor is fixed to the lower housing and measures the support-force adjustment-plate position $z_{p}$ . Plate velocity is obtained by filtering and differentiating this signal. Each instrumented foot contains an 18-cell distributed flexible thin-film plantar-pressure module; the 18 cells are not divided between the two feet. After calibration, each cell provides a local contact-force measurement. The system sums these signals to obtain total plantar pressure $F_{\text{foot}}$ and calculates heel pressure $F_{\text{heel}}$ , midfoot pressure $F_{\text{mid}}$ , forefoot pressure $F_{\text{fore}}$ , and COP. Spring support force is not measured directly. Its unloading-related effect is assessed indirectly from total and regional plantar pressure and changes in hip-knee loading (Ren and Liu, 2021; Conner et al., 2022; Liu et al., 2024; Cho, 2023; Jun et al., 2021; Ren et al., 2023). The measured variables and their roles in sensing, control, and evaluation are summarized in Table 1.

**TABLE 1:** Sensor configuration and function.

Sensor/signal	Measured variables	Main function
Multi-zone plantar-pressure sensor	$F_{1}$ - $F_{18}$ , $F_{\text{foot}}$ , $F_{\text{heel}}$ , $F_{\text{mid}}$ , $F_{\text{fore}}$ , COP	Gait-phase recognition, pressure closed-loop control, impact metrics, and load-transfer analysis
Hip/knee dual encoders	$q_{h}$ , $q_{k}$ , ${\dot{q}}_{h}$ , ${\dot{q}}_{k}$	Joint-state estimation and support for torque and energy analysis
Hip/knee voltage/current	$U_{\text{hip}}$ , $I_{\text{hip}}$ , $U_{\text{knee}}$ , $I_{\text{knee}}$	Evaluation of hip-knee power, measured actuator electrical energy, and load changes
Laser sensor for adjustment-plate position	$z_{p}$ , ${\dot{z}}_{p}$	Vertical-axis position loop and motion constraint of the support-force adjustment plate
Vertical-axis voltage/current	$U_{z}$ , $I_{z}$	Evaluation of additional actuator electrical energy from the vertical support mechanism

The main commercial components are identified to support replicability. The plantar-pressure sensor was a distributed flexible thin-film array acquisition module supplied by Guantuo Electronics (Hefei, Anhui, China). The hip and knee actuators were joint modules supplied by Hangzhou ChengTian Technology Development Co., Ltd. (Hangzhou, China). Adjustment-plate position was measured with a laser infrared ranging sensor module supplied by Jingxun Changtong (Weihai, China). These three components were supplied as integrated modules without separate commercial model designations; therefore, their product categories and suppliers are reported. The vertical actuator was an STM57M integrated stepper-servo motor supplied by NiMotion (Nanjing, China).

This configuration measures foot-ground contact, adjustment-plate motion, and electrical energy in each actuator channel. Because the spring’s upper attachment moves with the human-exoskeleton structure, the control law uses measurable plantar pressure rather than an unmeasured spring-force state. Total and regional plantar pressure and COP describe the resulting foot-ground contact. Hip-knee joint torque and measured actuator electrical energy quantify changes in joint loading and drive consumption. These measurements form the control chain from plantar-pressure feedback to adjustment-plate position control and actuator-energy evaluation.

### 2.3 Data acquisition and signal processing

#### 2.3.1 Signal acquisition, synchronization, and calibration

The acquisition system records plantar pressure, joint angles, motor voltage/current, and adjustment-plate position on a common time base. Plantar pressure, hip/knee encoder signals, and all motor voltage/current signals were sampled at 100 Hz. The laser distance signal was sampled at 50 Hz. During online control, the latest valid laser measurement was held on the 100-Hz controller time base until the next sample arrived. For offline analysis and figure generation, this signal was linearly resampled to 100 Hz. The online controller therefore used no future samples or non-causal interpolation. All channels were zeroed before each experiment. The plantar-pressure sensor was calibrated under no-load and known-load conditions. The laser sensor was calibrated linearly using the mechanical travel limits and known displacement points. The calibrated local contact force from pressure cell j is denoted by $F_{j}\left(t\right)$ . Total and regional plantar pressures are obtained by summing the corresponding cells (Ren and Liu, 2021; Conner et al., 2022).

#### 2.3.2 Plantar-pressure fusion and COP calculation

The total plantar force, regional plantar forces, and COP were calculated using Equations 1–3, respectively.

$$F_{\text{foot}}\left(t\right)=\overset{18}{\underset{j=1}{\sum }}F_{j}\left(t\right)$$ (1)

$$F_{r}\left(t\right)=\underset{j\in \Omega_{r}}{\sum }F_{j}\left(t\right),\;r\in \left\{heel,mid,fore\right\}$$ (2)

$$r_{\text{COP}}\left(t\right)=\frac{\sum_{j=1}^{18}F_{j}\left(t\right)r_{j}}{F_{\text{foot}}\left(t\right)}$$ (3)

Here, $F_{j}\left(t\right)$ is the calibrated contact force measured by the j -th plantar-pressure cell, with j=1,…,18 . $F_{\text{foot}}\left(t\right)$ denotes the total plantar force of the tested side. $F_{r}\left(t\right)$ denotes the regional plantar force, and $\Omega_{r}$ is the set of pressure cells assigned to the region r , where r represents the heel, midfoot, or forefoot region. The vector $r_{j}={\left[x_{j},y_{j}\right]}^{T}$ is the planar coordinate of the center of the j -th pressure cell in the foot-fixed insole coordinate system. The origin is located at the midpoint of the posterior edge of the effective sensing area, the x -axis is directed anteriorly along the longitudinal axis of the insole, and the y -axis is directed laterally. The vector $r_{\text{COP}}\left(t\right)={\left[x_{\text{COP}}\left(t\right),y_{\text{COP}}\left(t\right)\right]}^{T}$ denotes the center of pressure and is calculated only when $F_{\text{foot}}\left(t\right)$ is above the contact threshold.

#### 2.3.3 Filtering and gait-phase processing

Plantar-pressure signals are used for gait-phase recognition and pressure closed-loop control, so both response speed and noise robustness are required. Each pressure-cell signal is first low-pass filtered, and $F_{\text{foot}}$ , $F_{\text{heel}}$ , $F_{\text{mid}}$ , $F_{\text{fore}}$ , and COP are then calculated. Gait-phase recognition is based mainly on the total plantar-pressure threshold, pressure change rate, and regional load-transfer features. A rapid increase in heel pressure corresponds to initial contact; combined heel and midfoot loading corresponds to loading response; stable midfoot/forefoot loading corresponds to single support; and decreasing forefoot pressure approaching the threshold corresponds to toe-off (Liu et al., 2024; Ren et al., 2023; Zhang et al., 2022; Xia et al., 2023). The support-force adjustment-plate position is processed with median filtering to suppress occasional jumps, and its velocity is calculated using low-pass filtering and central differentiation. Plate acceleration is used only for motion-safety monitoring and is not used to calculate spring support force.

The filtering parameters were selected according to the characteristics of low-speed rehabilitation gait. Plantar-pressure signals were processed using a fourth-order low-pass Butterworth filter with a cutoff frequency of 12 Hz to preserve the main variations during contact and toe-off. Joint-angle signals were processed using an 8-Hz low-pass Butterworth filter, and angular velocity was calculated by numerical differentiation after filtering. Motor-current signals were processed using a 20-Hz low-pass Butterworth filter for torque and electrical-energy analysis. The laser distance signal was processed by a three-point median filter followed by a 6-Hz low-pass Butterworth filter before differentiation. Offline figure generation used zero-phase filtering, whereas real-time control used causal filtering with the same cutoff frequencies to prevent future data from entering the controller (Liu et al., 2024; Cho, 2023; Jun et al., 2021; Ren et al., 2023).

### 2.3.4 Hardware control and data flow

**FIGURE 3:**
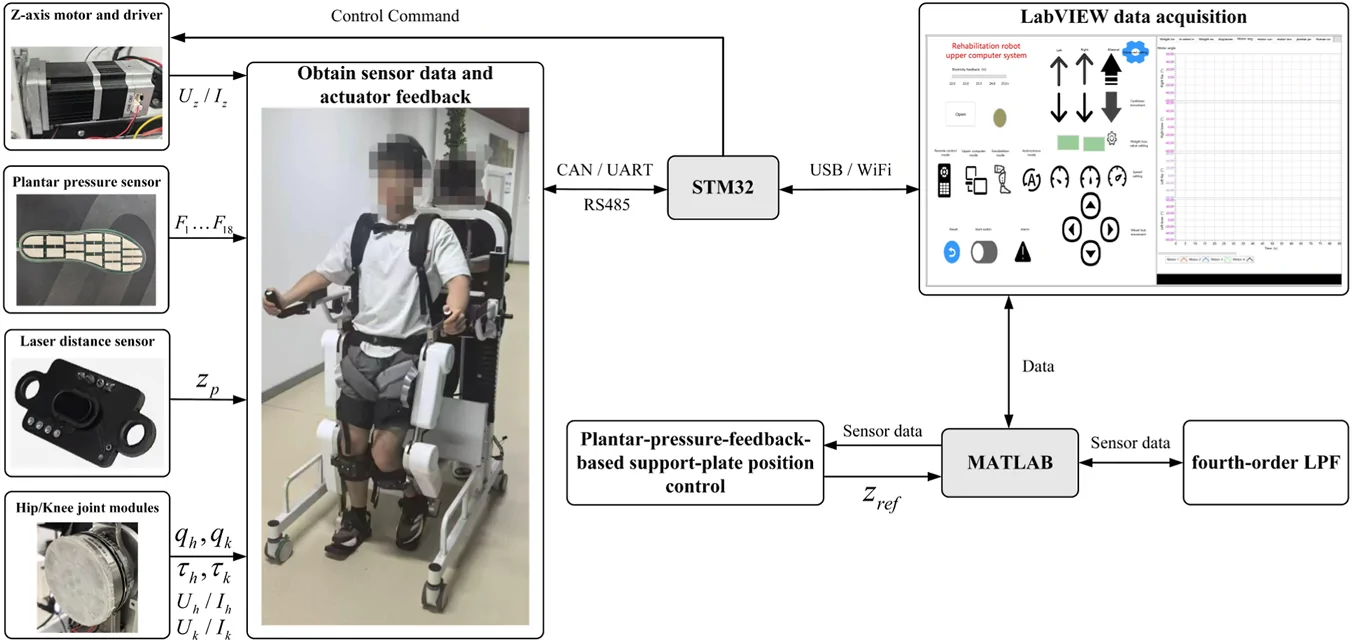
Hardware control and data-flow architecture of the exoskeleton. The STM32 controller acquires signals from the plantar-pressure insole, laser displacement sensor, hip/knee modules, and voltage/current channels. MATLAB and LabVIEW support filtering, pressure-zone fusion, gait-phase recognition, vertical-axis position-command generation, display, logging, and actuator-energy evaluation.

Figure 3 summarizes the hardware and data-flow architecture linking the robot, controller, upper-computer software, and processing modules. The 18-channel plantar-pressure insole provides $F_{1}$ - $F_{18}$ , and the laser sensor provides $z_{p}$ . The hip/knee modules provide $q_{h}$ , $q_{k}$ , torque-related signals, voltage, and current. The vertical-axis channel supplies voltage/current feedback and receives the position command. These signals reach the STM32 controller through CAN, UART, RS485, or analog acquisition links. The STM32 handles low-level acquisition, communication, and execution of the vertical-axis position loop. MATLAB performs filtering, pressure-zone fusion, gait-phase recognition, target-band calculation, position-command generation, and actuator-energy evaluation. LabVIEW provides the display and logging interface. Accordingly, Figure 3 traces the complete control path from sensor measurements to contact-state variables, target position, and low-frequency vertical-axis adjustment.

Hip and knee joint torque is obtained from the internal module estimate or approximated from motor current. When the module current constant $K_{t}$ is available, joint output torque is written as $\tau_{i}=K_{t}I_{i}\eta_{i}$ , where $\eta_{i}$ denotes the transmission efficiency or calibration coefficient. Electrical energy is calculated from voltage and current measured at each drive channel and is therefore reported as measured actuator electrical energy. The non-negative electrical-power metric $P_{i}\left(t\right)=U_{i}\left(t\right)|I_{i}\left(t\right)|$ is integrated over each gait cycle. Hip, knee, vertical-axis, and measured total actuator electrical energies are reported separately (Wang et al., 2022a; Woo et al., 2021; Lee and Rosen, 2023; Andersson and Bjorsell, 2022; Tang et al., 2022; Chen et al., 2025).

$$P_{i}\left(t\right)=U_{i}\left(t\right)|I_{i}\left(t\right)|,\;E_{i}=\int_{0}^{T}P_{i}\left(t\right)\;dt$$ (4)

$$E_{\text{act}}=E_{\text{hip}}+E_{\text{knee}}+E_{z}$$ (5)

In Eqs. Equation 4; Equation 5, i denotes the actuator channel, including the hip, knee, or vertical-axis actuator. $U_{i}\left(t\right)$ and $I_{i}\left(t\right)$ denote the measured drive-channel voltage and current, respectively. The absolute current is used because regenerative braking and driver-side energy recovery are not separately measured. T is the duration of one normalized gait cycle, $E_{i}$ is the corresponding actuator electrical energy per gait cycle, and $E_{\text{act}}$ is the measured total actuator electrical energy of the recorded hip, knee, and vertical-axis actuator channels.

### 2.4 Plantar-pressure-feedback-based support-force adjustment-plate position control

#### 2.4.1 Control objectives

The controller adjusts the target position of the support-force adjustment plate according to gait phase. Its objective is to keep tested-side total plantar pressure $F_{\text{foot}}$ within the phase-related target band while reducing initial-contact impact and stance-phase hip-knee loading. Here, $F_{\text{foot}}$ is the unilateral plantar contact force obtained by summing the calibrated outputs of the 18 cells on the tested side. When both feet are instrumented, the two sides can be calculated and controlled separately. The normalization reference BW is the participant’s body weight $m_{s}g$ . It excludes the walker frame, exoskeleton weight borne by the frame, and any external assistance from experimenters. Thus, $F_{\text{foot}}/BW$ supports relative comparisons across participants and conditions but does not represent the complete vertical force balance of the human-exoskeleton-walker system. During unilateral support or low-speed dynamic load transfer, its peak can reasonably approach or slightly exceed 1.

The numerical ranges in Table 2 were defined before the formal A/B/C comparison as engineering design intervals for the low-speed walker-assisted task. They were not tuned using the outcome data. Published body-weight-normalized stance-phase curves provided the phase-dependent shape and approximate reference loading envelope. This envelope included rapid loading after initial contact, sustained support-phase loading, and unloading before toe-off (Wolff et al., 2023; Wolff et al., 2024). A retained plantar-load coefficient of η=0.70 provided the nominal scaling. In a previous body-weight-support experiment, 30% unloading produced plantar forces equal to 68±4 % of unsupported walking, which did not differ significantly from the prescribed 70% retained load (Richter et al., 2021). This evidence informed the overall loading magnitude rather than the exact boundary for each gait phase.

**TABLE 2:** Gait-phase-related plantar-pressure target ranges.

Gait phase	Recognition basis	Target range of $F_{\text{foot}}/BW$
Swing	Target foot pressure below the contact threshold	0.00–0.05
Initial contact	Pressure rises rapidly and exceeds the contact threshold	0.05–0.30
Loading response	Pressure continues to rise and enters load transfer	0.30–0.70
Single support	Unilateral pressure dominates and remains relatively stable	0.50–0.80
Toe-off	Forefoot pressure decreases and approaches toe-off	0.15–0.55

The phase-specific limits were then rounded outward to the nearest 0.05 BW to accommodate inter-cycle variability and provide control margin. The contact threshold was 0.05 BW, corresponding to 32.9, 36.8, and 46.6 N for S1, S2, and S3, respectively. Nonzero stance-phase loading was retained to avoid excessive unloading. Wider loading-response and toe-off intervals accommodated transient transfer between plantar regions. This procedure yielded target bands of 0.05–0.30 BW for initial contact, 0.30–0.70 BW for loading response, 0.50–0.80 BW for single support, and 0.15–0.55 BW for toe-off. These values are task-specific engineering constraints rather than universal physiological or clinical reference ranges.

The ranges in Table 2 are not intended to reproduce the full vertical ground-reaction-force magnitude of normal overground walking. During normal walking, vertical ground reaction force can exceed body weight, particularly during weight acceptance and late-stance propulsion. In the present task, the walker-assisted exoskeleton and spring-supported structure bear part of the load. The controller therefore seeks to attenuate excessive contact impact without eliminating measurable foot-ground contact. The same target ranges were used for all participants and remained unchanged throughout the formal experiments.

$$F_{\min}\left(\phi \right)\leq F_{\text{foot}}\left(t\right)/BW\leq F_{\max}\left(\phi \right)$$ (6)

$$e_{F}\left(t\right)=0,\;F_{\min}\left(\phi \right)\leq F_{\text{foot}}\left(t\right)/BW\leq F_{\max}\left(\phi \right)$$ (7)

$$e_{F}\left(t\right)=F_{\text{foot}}\left(t\right)/BW-F_{\max}\left(\phi \right),\;F_{\text{foot}}\left(t\right)/BW>F_{\max}\left(\phi \right)$$ (8)

$$e_{F}\left(t\right)=F_{\text{foot}}\left(t\right)/BW-F_{\min}\left(\phi \right),\;F_{\text{foot}}\left(t\right)/BW<F_{\min}\left(\phi \right)$$ (9)

In Equations 6–9, ϕ denotes the current gait phase. $F_{\min}\left(\phi \right)$ and $F_{\max}\left(\phi \right)$ denote the lower and upper bounds of the phase-related plantar-pressure target band, respectively. BW denotes the participant body weight used for normalization. The variable $e_{F}\left(t\right)$ is the dead-zone pressure error. It is set to zero when $F_{\text{foot}}\left(t\right)/BW$ lies inside the target band, becomes positive when the normalized pressure exceeds the upper bound, and becomes negative when the normalized pressure is below the lower bound.

$$\Delta z_{\text{comp}}\left(t\right)=Comp\left(X_{t}\right)$$ (10)

$$z_{\text{ref}}=clip\left[z_{p}+K_{p}e_{F}+K_{i}\int e_{F}\;dt+\Delta z_{\text{comp}},\;z_{\min},\;z_{\max}\right]$$ (11)

$$z_{\min}\leq z_{\text{ref}}\leq z_{\max},\;|{\dot{z}}_{p}|\leq {\dot{z}}_{\max},\;F_{\text{foot}}/BW\geq F_{\text{safe}}$$ (12)

In Equations 10–12, $X_{t}$ denotes the short-time fused state window used by the compensation branch. It contains plantar-pressure zone features, COP, gait phase, support-force adjustment-plate position $z_{p}$ , plate velocity ${\dot{z}}_{p}$ , and hip/knee joint states. Comp(⋅) denotes the small temporal compensation component, and $\Delta z_{\text{comp}}\left(t\right)$ is its bounded position correction. The reference position $z_{\text{ref}}$ is obtained by adding the measured plate position $z_{p}$ , the dead-zone PI correction with gains $K_{p}$ and $K_{i}$ , and the compensation term. The operator clip[⋅] restricts the command within the physical travel limits $z_{\min}$ and $z_{\max}$ . The safety constraints limit the commanded position, measured plate velocity ${\dot{z}}_{p}$ , and minimum normalized plantar contact force $F_{\text{safe}}$ . In the experiments, $z_{\min}=0$ mm and $z_{\max}=80$ mm were selected according to the mechanical travel of the support-force adjustment plate, ${\dot{z}}_{\max}=20$ mm/s was used to keep the adjustment slow relative to the gait cycle, and $F_{\text{safe}}=0.05$ BW was used as the minimum contact threshold during stance-related phases.

#### 2.4.2 Support-force adjustment-plate position control

The controller uses support-force adjustment-plate position $z_{p}$ as its output and total plantar-pressure range error as feedback. When $F_{\text{foot}}/BW$ exceeds the upper bound for the current gait phase, the plate moves upward. This motion tends to increase the working compression of the fixed-stiffness spring and raise the unloading-related support working point. When the normalized pressure falls below the lower bound, the plate moves downward, reducing the unloading-related working point and restoring foot-ground contact. The closed loop neither measures nor precisely controls spring force. Instead, plantar-pressure-feedback-based plate positioning constrains the measurable foot-ground contact load.

The baseline controller uses a dead-zone PI term to generate a low-frequency correction of support-force adjustment-plate position. A small temporal compensation component follows the PI output to accommodate interparticipant differences in body weight and short-term variation in harness condition and gait pattern. This component uses a gated recurrent unit (GRU) only to predict a bounded position correction. It does not alter the target-band-centered control structure and is not evaluated as an independent contribution. The single-time-step input vector contains $F_{\text{foot}}/BW$ , regional pressures, COP, gait phase, $z_{p}$ , ${\dot{z}}_{p}$ , hip/knee joint angles, and hip/knee currents. The output is the plate-position compensation $\Delta z_{\text{comp}}$ .

The small temporal compensation component does not bypass the safety constraints. Its output is limited by the maximum displacement, maximum velocity, and plantar-pressure safety threshold. When sensor signals are abnormal or the pressure is lower than the minimum safe contact threshold, the controller returns to a conservative position-control mode. The vertical-axis motor velocity is limited to avoid discomfort or structural impact caused by rapid adjustment-plate motion.

The small temporal compensation component uses within-subject training. Preliminary trials from each participant provide the training samples. The label $\Delta z_{\text{comp}}$ is derived from the PI-control residual and an offline receding-horizon correction applied to the preliminary data. Each input window contains 10 samples on the 100-Hz control time base. The auxiliary model comprises one GRU layer with 64 hidden units, a dropout layer with probability 0.2, and fully connected layers with 32, 16, and 1 unit. Training uses the Adam optimizer, a learning rate of 0.001, a batch size of 32, and a maximum of 100 epochs. Complete trials, rather than adjacent gait cycles, are assigned to the training, validation, and test sets. The group C test trials are excluded from model training and hyperparameter selection. The component supplies only a small temporal correction; pressure feedback, the dead-zone PI term, and safety constraints jointly determine the final position command.

**FIGURE 4:**
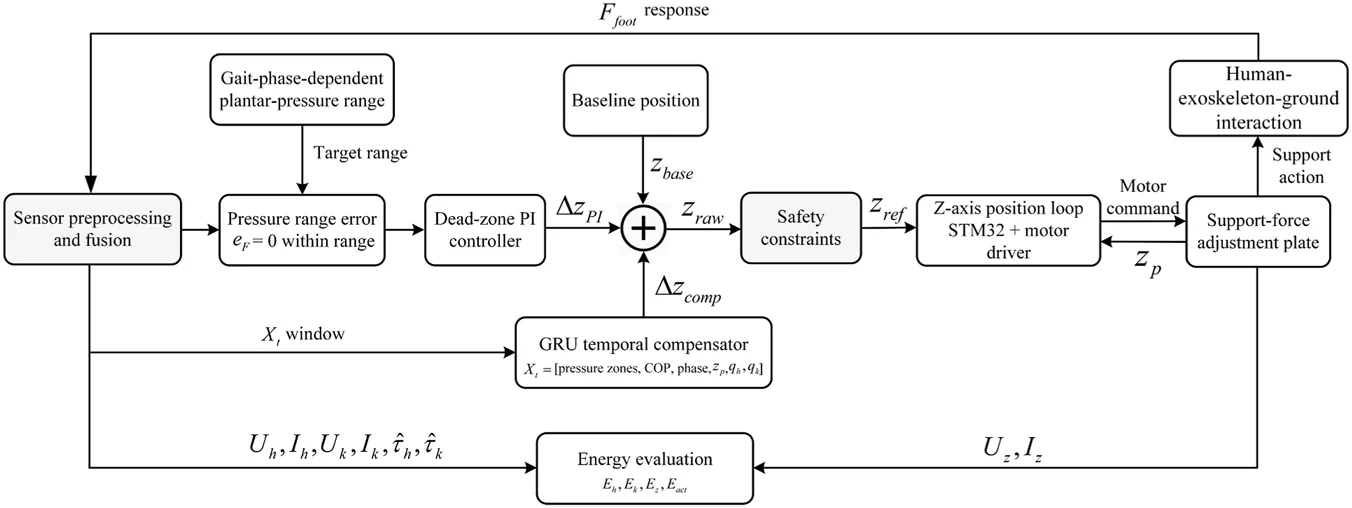
Control block diagram for plantar-pressure-feedback-based positioning of the support-force adjustment plate. The diagram includes the phase-related target band, pressure-range error, dead-zone PI controller, small temporal compensation branch, safety constraints, vertical-axis position loop, and measured plantar-pressure response.

The control algorithm proceeds as shown in Figure 4. First, sensor data are acquired and filtered, and total plantar pressure, regional pressures, and COP are calculated. Second, the current gait phase is recognized and the corresponding pressure target band is retrieved. Third, the dead-zone pressure error and small temporal compensation are calculated. Fourth, the vertical-axis target position is generated after position, velocity, and plantar-pressure safety constraints. Finally, the support-force adjustment-plate position loop is executed and energy metrics are recorded. The entire process uses measurable plantar-contact output as the outer-loop feedback variable.

### 2.5 Experimental protocol

#### 2.5.1 Experimental conditions

**FIGURE 5:**
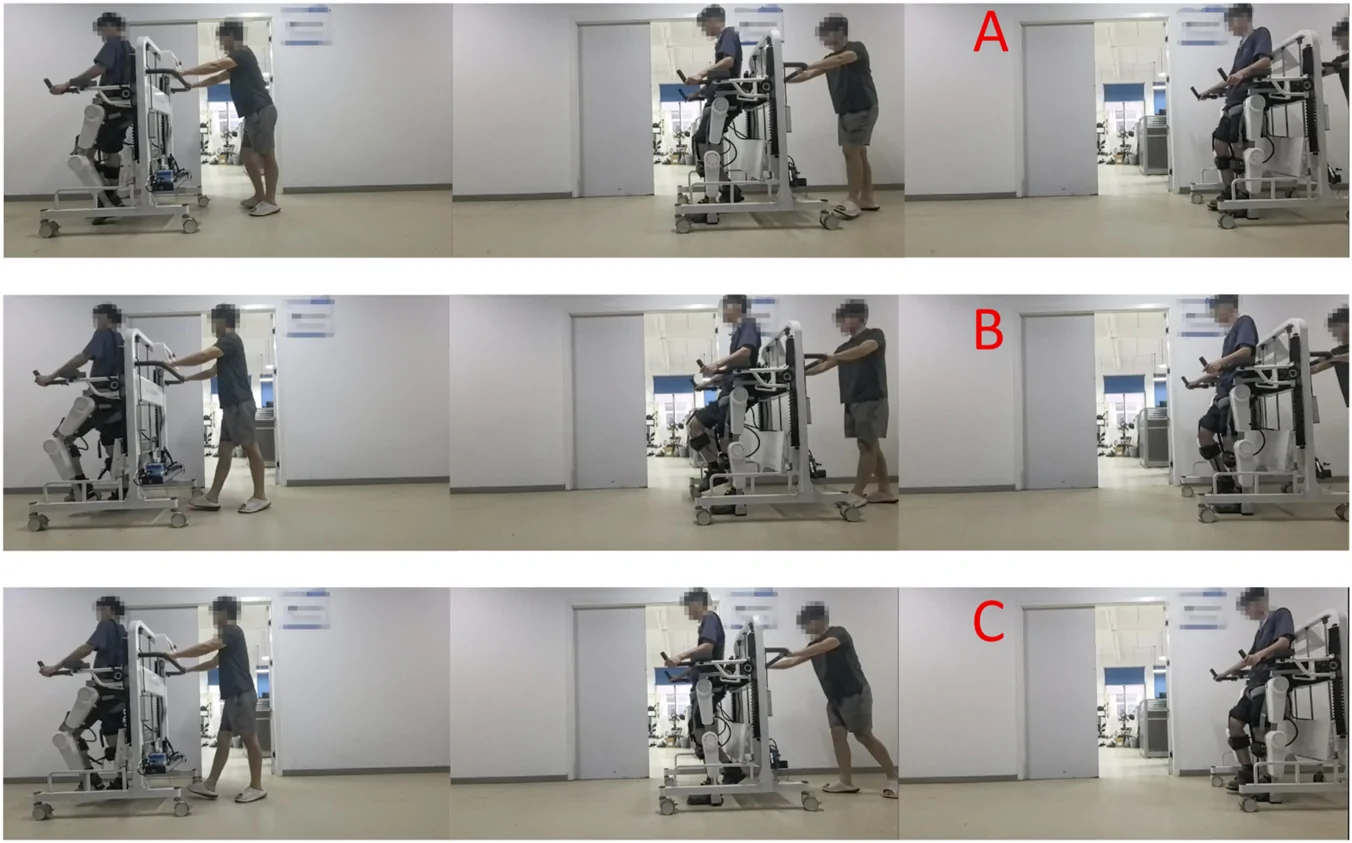
Experimental conditions and procedure. Conditions were **(A)** no spring, **(B)** passive spring, and **(C)** proposed method. The procedure included participant preparation, sensor calibration, adjustment-plate control settings, data acquisition, stable-cycle selection, and metric calculation.

Three conditions were used to evaluate the engineering feasibility of the proposed method (Figure 5). In group A (No spring), vertical-axis adjustment was disabled, the spring buffer was disconnected, and the support-force adjustment plate remained fixed. In group B (Passive spring), the plate remained fixed while the fixed-stiffness spring provided passive buffering. In group C (Proposed method), plantar-pressure-feedback-based plate-position control operated with the passive spring and the small temporal compensation component. Group B included neither vertical-axis position following nor active adjustment; its vertical-axis actuator electrical energy was therefore 0.

#### 2.5.2 Participants and familiarization

Three healthy male adults participated. S1 was 24 years old, 185 cm tall, and weighed 67 kg; S2 was 25 years old, 178 cm tall, and weighed 75 kg; S3 was 24 years old, 182 cm tall, and weighed 95 kg. Participants were eligible if they were at least 18 years old, could walk independently, understood the experimental instructions, and fitted within the exoskeleton’s adjustable dimensions. They also reported no neurological, neuromuscular, or musculoskeletal condition affecting gait. Exclusion criteria included current lower-limb injury or pain, skin damage at human–robot contact sites, a contraindication to device-assisted walking, or inability to obtain a safe exoskeleton fit. Testing was also discontinued in response to discomfort, excessive fatigue, or another safety-related event. All participants provided written informed consent.

All participants were members of the robot-development team and had prior experience operating and wearing the exoskeleton. Before formal data collection, each completed at least five familiarization trials to establish stable low-speed frame-assisted walking and verify sensor operation.

#### 2.5.3 Testing procedure and cycle selection

Each participant completed five walking trials per condition. Conditions were not randomized; all participants followed the same predefined order to maintain a consistent hardware setup and spring-connection procedure. Rest intervals were provided between conditions. The measured walking speed was approximately 0.18 m/s. This speed reflected the low-speed walker-assisted operating condition of the prototype, which prioritized safety, stable foot-ground contact, and reliable support-plate adjustment over normal overground walking speed.

After start and stop segments were removed, 6–10 consecutive stable gait cycles from each trial were retained for analysis. Eligible cycles had continuous foot-pressure acquisition, no saturation or signal dropout, no emergency stop, no environmental collision, and no visually identifiable external traction from the experimenter. Selection did not consider outcome values such as pressure peak, loading rate, COP offset, joint torque, or actuator energy. The experimenter provided safety protection only, preventing path deviation or collision without actively pulling the participant or walker. All analyzed cycles therefore came from stable walking segments with no identifiable external traction.

#### 2.5.4 Cycle normalization and averaging

Plantar pressure, joint torque, voltage/current, and adjustment-plate position were aligned by timestamp. Initial contact was identified from the tested-side total plantar-pressure threshold and rising edge. Each stable gait cycle was then normalized to 101 samples. Cycles were first averaged within each participant and condition; means and standard deviations were subsequently calculated across the three participants.

### 2.6 Evaluation metrics

The plantar-pressure metrics were calculated from the tested-side normalized total plantar pressure $F_{\text{foot}}/BW$ . The five plantar-pressure evaluation metrics are defined in Equations 13–17. The peak pressure, loading rate, out-of-band duration, and initial-contact impact impulse were defined as

$$F_{\text{peak}}=\underset{t\in T_{\text{gait}}}{\max}\frac{F_{\text{foot}}\left(t\right)}{BW},$$ (13)

$$LR=\underset{t\in T_{\text{IC}}}{\max}\frac{d}{dt}\left(\frac{F_{\text{foot}}\left(t\right)}{BW}\right),$$ (14)

$$D_{\text{out}}=\frac{1}{T_{\text{gait}}}\int_{T_{\text{gait}}}I\left[\frac{F_{\text{foot}}\left(t\right)}{BW}<F_{\min}\left(\phi \right)\;or\;\frac{F_{\text{foot}}\left(t\right)}{BW}>F_{\max}\left(\phi \right)\right]dt\times 100\%,$$ (15)

$$J_{\text{IC}}=\int_{T_{\text{IC}}}\frac{F_{\text{foot}}\left(t\right)}{BW}\;dt.$$ (16)

Here, $T_{\text{gait}}$ denotes one gait cycle, $T_{\text{IC}}$ denotes the 0–200 m initial-contact window after heel contact, and I[⋅] is an indicator function. $F_{\text{peak}}$ is dimensionless, LR is reported in BW/s, $D_{\text{out}}$ is reported as the percentage of gait-cycle duration, and $J_{\text{IC}}$ is reported in BW ⋅ s. The lateral COP offset was defined in the foot-fixed coordinate system as

$$D_{\text{COP}}=\frac{1}{N_{\text{st}}}\overset{N_{\text{st}}}{\underset{n=1}{\sum }}|y_{\text{COP}}\left(t_{n}\right)-y_{0}|,$$ (17)

where $y_{\text{COP}}$ is the medial-lateral COP coordinate, $y_{0}$ is the centerline of the effective sensing area, and $N_{\text{st}}$ is the number of stance samples. $D_{\text{COP}}$ is reported in mm without foot-width normalization. Because the primary analysis involved within-participant comparisons across conditions A–C, the same pressure insole, effective sensing-area geometry, footwear, and sensor-alignment procedure were maintained for each participant across all conditions. Shoe size was not used as a surrogate for measured foot width. Joint-load metrics were represented by the absolute peak hip and knee torques. Electrical-energy metrics were represented by $E_{\text{hip}}+E_{\text{knee}}$ , $E_{z}$ , and $E_{\text{act}}$ as defined in Equation 4 and Equation 5.

### 2.7 Statistical analysis

This preliminary engineering feasibility experiment involved three healthy male participants, so the analysis was descriptive rather than inferential. For each participant and condition, stable gait cycles were averaged within trials and then across trials. Group values are reported as the mean and standard deviation across participants. Cycle-level samples were not treated as independent observations for hypothesis testing because adjacent cycles from the same participant are correlated. Accordingly, no population-level statistical significance is claimed. Condition effects were assessed from the direction and magnitude of participant-level changes, consistency across individuals, and agreement among plantar-pressure, joint-torque, and measured actuator-energy metrics.

## 3 Results

### 3.1 Plantar-pressure control results

**FIGURE 6:**
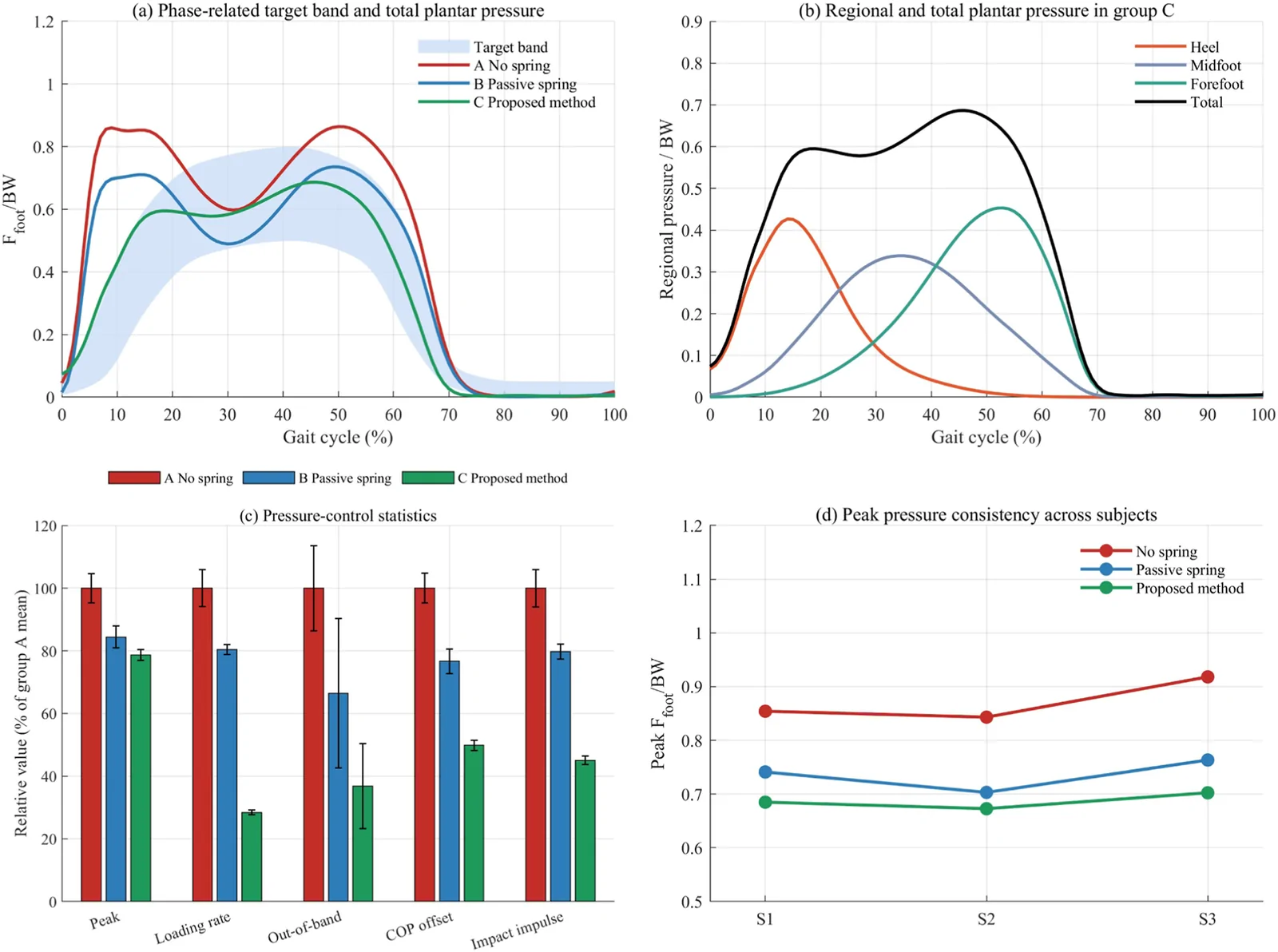
Plantar-pressure responses and pressure-control metrics. **(a)** Phase-related target band and tested-side total plantar pressure $F_{\text{foot}}/BW$ under **(A)** no spring, **(B)** passive spring, and **(C)** proposed method. **(b)** Heel, midfoot, forefoot, and total plantar-pressure curves in group C. **(c)** Peak pressure, loading rate, out-of-band duration, lateral COP offset, and initial-contact impact impulse. **(d)** Peak-pressure consistency across S1, S2, and S3. Curves were derived from measured data by low-pass filtering, stable gait-cycle segmentation, normalization to 0%–100% of the gait cycle, and multi-cycle averaging. Shading denotes the target band, and error bars in **(c)** show between-subject dispersion.

Figure 6 shows the plantar-pressure results for the three participants. In Figure 6a, group A had higher total plantar-pressure peaks during loading response and before toe-off. Passive spring buffering reduced pressure in group B, although the response remained outside the target band during part of the gait cycle. Group C remained generally closer to the phase-related target band and exhibited a smoother initial-contact pressure rise. Across participants, peak total plantar pressure in group C was 0.687±0.015 BW, 21.2% lower than in group A ( 0.872±0.041 BW) and 6.7% lower than in group B ( 0.736±0.030 BW). Loading rate decreased from 9.02±0.53 BW/s in group A and 7.25±0.14 BW/s in group B to 2.56±0.07 BW/s in group C, reductions of 71.6% and 64.7%, respectively. Initial-contact impact impulse decreased from 0.143±0.009 BW ⋅ s in group A and 0.114±0.003 BW ⋅ s in group B to 0.064±0.002 BW ⋅ s in group C. In group C, the heel, midfoot, and forefoot regions contributed sequentially to weight bearing, with continuous load transfer between regions (Figure 6b). The out-of-band duration was 18.81±6.93 %, and lateral COP offset was 11.03±0.37 mm, both lower than in groups A and B (Figure 6c). Individual results in Figure 6d show the same peak-pressure order, A>B>C , for S1, S2, and S3.

### 3.2 Hip-knee joint load and energy

**FIGURE 7:**
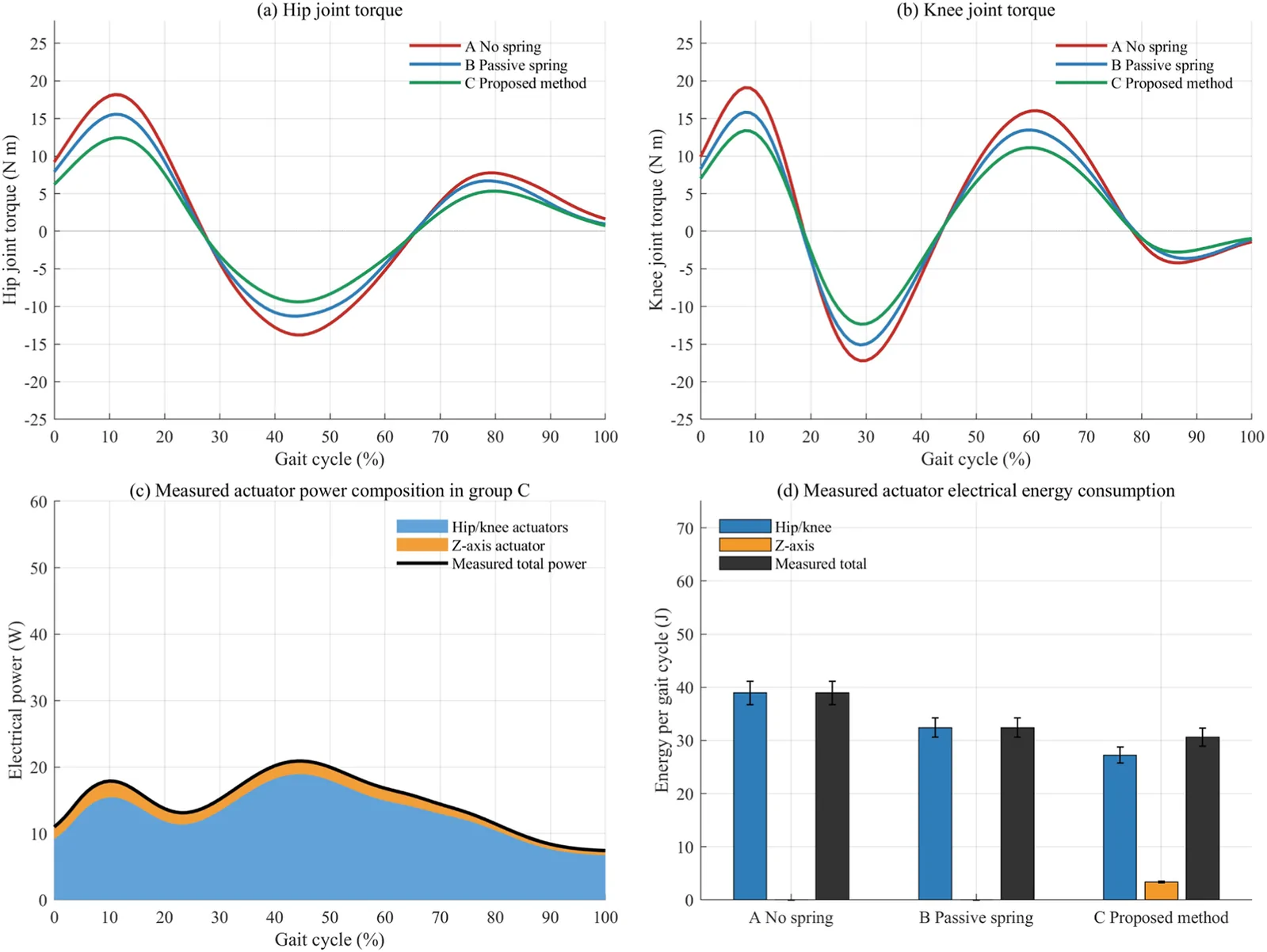
Hip-knee joint torque and measured actuator electrical-energy responses. **(a)** Hip joint torque. **(b)** Knee joint torque. **(c)** Hip-knee actuator power, vertical-axis actuator power, and measured total actuator power in group C. **(d)** Hip-knee, vertical-axis, and measured total actuator electrical energy. Torque curves are multi-cycle averages after time normalization; signs follow the coordinate convention of the joint modules.

Figure 7 presents hip-knee joint torque and measured actuator electrical energy. Around the main stance-phase torque peaks, group C had lower hip and knee torque amplitudes than groups A and B (Figures 7a,b). Across participants, peak absolute hip torque decreased from 18.19±1.07 N ⋅ m in group A to 12.46±0.30 N ⋅ m in group C. Peak absolute knee torque decreased from 19.09±1.08 to 13.38±0.67 N ⋅ m. Hip-knee actuator electrical energy was 38.95±2.20 , 32.41±1.83 , and 27.24±1.54 J/gait cycle in groups A, B, and C, respectively. Group C was 30.1% lower than group A and 15.9% lower than group B. Because the support-force adjustment plate remained fixed in groups A and B, vertical-axis active-adjustment energy was 0. In group C, vertical-axis actuator electrical energy was 3.37±0.16 J/gait cycle. Including this term gave a measured total actuator electrical energy of 30.60±1.68 J/gait cycle, 21.4% lower than group A and 5.6% lower than group B. Peak hip-knee and vertical-axis actuator powers in group C were approximately 18.92 W and 2.46 W, respectively (Figure 7c). Thus, although the vertical-axis channel contributed a relatively small additional energy term, it was included in the actuator-energy evaluation.

## 4 Discussion

The proposed position-control method altered plantar contact in two related ways. First, the pressure-band constraint produced a smoother initial-contact pressure rise in group C. Loading rate and impact impulse decreased more than peak pressure, suggesting that the response was not simply a uniform reduction in foot-ground loading. Transient impact was attenuated while plantar loading and frictional contact were retained. This distinction matters because excessively low plantar pressure can weaken the load-transfer information available from stance-phase pressure measurements (Wolff et al., 2023; Conner et al., 2022; Ren et al., 2023). Second, multi-zone pressure fusion and COP estimation captured both load magnitude and location. The continuous heel-midfoot-forefoot transition in Figure 6b is consistent with a phase-related target band rather than a constant pressure set point.

The energy results clarify the contribution of the support-force adjustment-plate mechanism. Passive spring buffering in group B reduced plantar pressure and hip-knee actuator energy relative to group A but could not correct pressure deviations across gait phases. Group C added vertical-axis actuation, which introduced a measurable electrical-energy cost. Reporting only the hip-knee reduction would therefore overstate the energy benefit. Here, actuator energy comprises the measured electrical energy of the hip, knee, and vertical-axis drive channels. After vertical-axis energy was included, group C still had lower measured total actuator electrical energy than groups A and B (Figure 7d). The vertical-axis actuator also remained a low-frequency, low-power channel (Figure 7c). Together, Figures 6, 7 show that the reduction in hip-knee actuator effort exceeded the additional vertical-axis energy cost under the tested conditions.

These results apply to low-speed frame-assisted walking in three healthy male participants. The tested speed of 0.18 m/s allowed stable prototype operation and sufficient time for low-frequency support-plate adjustment. At higher speeds, shorter gait phases and the delay and speed limits of the screw-driven actuator may reduce the available adjustment time. Faster operation would require evaluation of actuator bandwidth, gait-phase estimation delay, position-rate constraints, and potentially a more predictive control strategy. In users with neurological impairment, altered plantar sensation may change loading behavior and its relationship to intended foot-ground contact. Asymmetric loading may require side-specific target bands and controller parameters, while spasticity may produce rapid, irregular load transitions. Inconsistent foot placement may also affect regional pressure and COP estimation. Finally, the fixed, non-randomized condition order may have introduced familiarization or order effects. The present experiment therefore evaluates controller behavior in a controlled low-speed setting rather than clinical rehabilitation efficacy.

The support mechanism was evaluated from measurable outputs rather than direct spring-force measurements. Because the spring’s upper attachment moves with the human-exoskeleton structure, adjustment-plate position alone does not uniquely determine instantaneous spring compression. Plantar pressure therefore served as the outer-loop feedback variable. Unloading-related effects were assessed from total plantar pressure, regional load transfer, COP, hip-knee torque, and actuator electrical energy. The energy boundary was defined explicitly: $P_{i}=U_{i}|I_{i}|$ represents electrical power measured at the recorded drive channels. It excludes regenerative recovery, spring energy storage and release, controller electronics, sensors, upper-computer power, and human metabolic energy. Passive spring storage and release may have contributed to actuator-load differences among conditions. The energy results therefore compare only the recorded actuator electrical channels and do not represent net mechanical efficiency or whole-system energy consumption.

## 5 Conclusion

This study presented a multi-zone plantar-pressure-guided position control method for the support-force adjustment plate of a walker-assisted lower-limb exoskeleton. The method uses tested-side total plantar pressure as its main feedback variable, with regional pressures and COP providing auxiliary contact-state information. Adjustment-plate position is the control output. During low-speed walker-assisted walking with three healthy participants, the proposed method showed lower peak plantar pressure, loading rate, initial-contact impact impulse, lateral COP offset, and hip-knee actuator electrical energy than the comparison conditions. Measured total actuator electrical energy also remained lower after vertical-axis energy was included. These preliminary findings support the engineering feasibility of plantar-pressure-feedback-based adjustment-plate position control for attenuating initial-contact impact and reducing measured actuator load while maintaining continuous plantar contact.

## Data Availability

The raw data supporting the conclusions of this article will be made available by the authors, without undue reservation.
